# Editorial: Health promotion in schools, universities, workplaces, and communities

**DOI:** 10.3389/fpubh.2024.1528206

**Published:** 2024-12-05

**Authors:** Graça S. Carvalho, Teresa Vilaça

**Affiliations:** CIEC, University of Minho, Braga, Portugal

**Keywords:** health education, health promoting school, schools for health in Europe, workplace health promotion, university health promotion, health literacy

This editorial summarizes new developments in the Public Health Research Topic “*Health Promotion in Schools, Universities, Workplaces, and Communities”*, which are places and social contexts where people engage in daily activities and in which environmental, policy, organizational, and individual factors interact to affect health and wellbeing. This Research Topic was very appealing as 105 manuscripts were submitted, and, following a rigorous evaluation and improvements suggested by high-level reviewers, 44 (41,9%) papers were accepted for publication. Of these, 9 focus on health promotion in schools, 15 in universities, 5 in workplaces, and 15 in communities.

## 1 Health promotion in schools

Schools are recognized worldwide as key settings for health promotion (7), as they provide access to a vast population of children from an early age to develop health knowledge and healthy behaviors (1, 6), as well as to tackle social determinants of health inequality (8). The nine papers in this item are distributed in two issues.

### 1.1 Four papers look at global school health education and promotion

Walker et al. intended to better understand how components of a whole-of school approach are implemented in practice by qualitatively exploring physical activity approaches and quantitatively assess implementation levels in US elementary schools, and examined associations between school-level physical activity promotion and academic ratings; Darlington-Bernard et al., did a scoping review in English and French literature to identify definitions on Life Skills in the field of health promotion at school, aiming to propose a conceptual definition and to reach a conceptual and consensual definition; Hobusch et al. reported on the integration of One Health principles educational frameworks, particularly within the context of the Austrian teaching training approach “Teaching Clinic”, which aligns with the 2030 Agenda, Education for Sustainable Development, and the Sustainable Development Goals; and Gay et al. developed methodologies for collecting and analyzing mass qualitative data, enabling to measure how a cohort identifies and prioritizes preventive interventions to identify differences in conceptions of the determinants of health and cancer between girls and boys, as expressed and perceived by French school children and adolescents.

### 1.2 Five papers focused on specific topics of school health education and promotion

Yan et al. identified predictors of e-cigarette uptake in Chinese youths with no prior tobacco use, considering individual, familial and the broader societal environmental factors; Li and Cheong designed and developed a functional training programme (ADDIE) that can be incorporated into existing physical education lessons to improve Malaysian primary school students' physical fitness levels; Smith et al. described the Revved Up Kids (RUK) intervention in US that provides necessary tools for elementary-aged children recognize and avoid dangerous people and situations, and evaluated its components and documents effectiveness; Summers et al. elucidated how parents/guardians of elementary and middle school students in Maryland (USA) navigated the return to in-person school following remote instruction and understand how they perceived communication about school-based COVID-19 mitigation strategies and their preferences for the content and format of public health communication about COVID-19 mitigation in schools; and Hyde et al. examined the current state of social innovation and entrepreneurship programming, courses, and centers within US schools of public health, identifying key areas as opportunities for growth.

## 2 Health promotion in universities

Institutions of higher education are recognized as relevant settings to protect health and promote the well-being of the academic community (students, staff and the wider community) through their policies and practices and by linking health promotion to teaching and research (4, 5). Of the 15 papers in this item, 6 are on health topics related to higher education students, 5 on medical and nurse students' training and their health conditions, and 4 on situations related to COVID-19 in higher education.

### 2.1 Six papers look at health topics related to high education students

Heller et al. investigated, in Germany, which of the predominant health behaviors (physical activity, healthy diet, sleep, sedentary behavior, alcohol consumption, smoking, drug use) are most associated with academic performance and whether the personal resource of trait mindfulness moderates these associations; Ladd determined the status of Health, Wellness, Physical Fitness Lecture (HWPFL) and Health-Related Physical Activity (HRPA) courses in Texas community colleges; Aldhahir et al. evaluated the smoking prevalence among students on healthcare courses in Saudi Arabia; Lavilla-Gracia et al. estimated the effectiveness of the peer-led BASICS intervention to reduce risky alcohol consumption among university students in the Spanish context; Tang et al. explored the association between health information preferences and specific health behaviors and outcomes, such as preventive measures and chronic disease management among Chinese college students; and Alkhatib et al. evaluated the effectiveness of the traditional Chinese Baduanjin exercise on reducing the menstrual symptoms for international female students studying in China during the acculturation period.

### 2.2 Five papers focus on issues related to medical and nurse students' training and their health conditions

Jürgensen et al. did a scoping review to highlight the current scope of research on the health of health professional students; Ramón-Arbués et al. determined the prevalence of symptoms of depression and anxiety, and the association between these symptoms and health-related behaviors in a population of Spanish student nurses; Hwang and Kim examined the mediating effects of self-directed learning competency on the relationships between optimism, emotional intelligence and academic resilience among South Korea nursing students; Yang et al. verified the role of Chinese medical students' physical literacy on health-related quality of life and explored the chain mediating role of physical activity and subjective wellbeing in it; and Gea-Caballero et al. estimated the level of attitudinal change in nursing students for immigrants, based on a training intervention with sessions of coexistence with immigrants in Spain.

### 2.3 Four papers explore situations related to COVID-19 in higher education

Alwan et al. assessed hand hygiene awareness and practice levels among various university communities in Lebanon; Korn et al. estimated academic distress among Israel undergraduate students following COVID-19, characterized its nature regarding economic, social, and health indicators, and examined the level of request for help subsequent mental distress; Ahdut-HaCohen and Carmel examined the impact of Israel academic institutions on changes to students' awareness and habits regarding a healthy lifestyle, specifically through nutrition and physical exercise, following the Covid-19 pandemic; and Navas-Echazarreta et al. analyzed the differences in smartphone use and the prevalence of nomophobia according to gender and university degree of health sciences students at the University of Zaragoza, Spain, during the COVID-19 confinement.

## 3 Health promotion in workplaces

Workplace Health Promotion refers to policies and practices that encourage employers, employees and society to improve the health and wellbeing of people at work by improving the work organization and environment, promoting active employer and employee participation and encouraging personal development (2).

### 3.1 The five papers submitted to this item focused on health promotion in workplaces

Schaller et al. did a scoping review of reviews to obtain an overview of the main objectives and related outcomes of physical activity interventions in workplace health promotion, as well as the setting-specific aspects considered in the research field; Friedrich et al. investigated the association of occupational health literacy (individual level), health-oriented leadership (interpersonal level), participation possibilities in health, and values of health in German companies (both organizational levels) on work ability; Wang et al. assessed the level of health literacy and health investment intention among employees in one of China's largest petrochemical companies and explored the effect of health literacy on health investment intention; Brandt, Schwandner et al. investigated whether resistance exercise snacking could be an efficient training approach for the workplace health promotion to minimize barriers for participation and facilitate resistance training in German women to improve musculoskeletal fitness; and Brandt, Heinz et al. investigated whether CrossFit^®^ is an effective training concept to motivate German employees toward healthy behaviors to improve fitness and health in the long-term in inactive employees with sedentary occupations.

## 4 Health promotion in communities

Community health promotion seeks to enable “people to have control over their health and its determinants strengthens communities and improves lives” (8, p. 1). Ethnic minorities and some migrant communities suffer health inequalities disproportionately (8). Community participation is crucial to creating the conditions that empower people to have greater control over their health and wellbeing within their specific social context (3). The 15 papers in this item are equally distributed in the following three issues.

### 4.1 Five papers focus on community minorities, inequalities and inclusion

Kutchma et al. monitored the implementation of a programme on US vulnerable communities (Black, Hispanic, or low-income people) that included free community-based testing, screening for and assistance with social determinants of health, dissemination of relevant and reliable COVID-related information, provision of personal protective equipment, and facilitation of access to vaccines; Krishnan et al. assessed the feasibility of the crowdsourcing method to provide an alternative approach that can improve household waste segregation using an 'online-slogan-contest' in different geographic regions of India; Yao et al. examined perspectives of experienced Community Health Workers (CHWs) about how CHWs can be integrated and utilized in US school settings to work as part of school-based health teams to support student health and healthy school environments; Kohut et al. interviewed students of Hospital Lang Youth Medical Program (LYMP) participating for 10 years in a mentoring and enrichment program for underrepresented minority youth in Upper Manhattan (USA) to explore their perspectives on what aspects of the program had the most impact on their academic and career paths; and Ahmed et al. developed a pilot intervention in Norway aiming to improve health in migrant communities through meaningful integration of highly educated migrants in academic environments.

### 4.2 Five papers look at health topics related to nutrition, physical activity, and sexual and reproductive rights

Lafave et al. revised and updated the Canadian Behavior, Attitude and Nutrition Knowledge Survey (C-BANKS) to align with the current Canadian food guide and dietary guidance and also reported on its reliability and validity; Monsalves-Álvarez et al. developed community intervention strategies in Chile, using a participatory action research approach by identifying barriers and facilitators on the practice of healthy eating and physical activity habits; Qin et al. made a correction to the paper that looked at the effects of whole-body electromyostimulation training on upper limb muscles strength and body composition in moderately trained Chinese males; Arnaiz et al. evaluated the short-term effects of the physical and health KaziKidz intervention on cardiometabolic risk factors and the long-term, pre-and post-COVID-19 pandemic changes thereof in South African high-risk children from marginalized communities; and Jemberie et al. assessed husbands' knowledge, involvement and factors influencing their involvement in Ethiopian women's sexual and reproductive health rights.

### 4.3 Five papers explore health education and health literacy in communities

Onyinyechi et al. synthesized in a systematic review the evidence of the impact of health education on malaria knowledge and insecticide-treated nets (ITN) usage in sub-Saharan Africa; Zhao and Fu examined the role of language in discerning the authenticity of online health rumors in China to better understand the credibility of online health information; Tamayo-Fonseca et al. assessed the level of health literacy and analyzed its relationship with sociodemographic variables, state of health, and use of health services in the population aged 15 and over in the Valencian Community of Spain; Murakami et al. estimated the associations of combinations of general Health Literacy (HL) and COVID-19-related HL with COVID-19 protective behaviors and healthy lifestyle behaviors in Japanese metropolitan areas; and Luo et al. investigated the impact of cultural capital on health literacy during the COVID-19 pandemic among Chinese community residents and to further examine the mediating role of social capital in the relationship between cultural capital and health literacy.

The research presented in these 44 papers published in the Public Health Research Topic “*Health Promotion in Schools, Universities, Workplaces, and Communities”* came from 18 countries of the various continents, demonstrating how live and pertinent is this field of research worldwide, presenting different approaches and perspectives that enrich this large, contemporary and relevant research field, which is still open to wide-ranging and diverse future research.
